# Neural Network-Based Muscle Torque Estimation Using Mechanomyography During Electrically-Evoked Knee Extension and Standing in Spinal Cord Injury

**DOI:** 10.3389/fnbot.2018.00050

**Published:** 2018-08-10

**Authors:** Muhammad Afiq Dzulkifli, Nur Azah Hamzaid, Glen M. Davis, Nazirah Hasnan

**Affiliations:** ^1^Department of Biomedical Engineering, Faculty of Engineering, University of Malaya, Kuala Lumpur, Malaysia; ^2^Discipline of Exercise and Sports Sciences, Faculty of Health Sciences, The University of Sydney, Sydney, NSW, Australia; ^3^Department of Rehabilitation Medicine, Faculty of Medicine, University of Malaya, Kuala Lumpur, Malaysia

**Keywords:** functional electrical stimulation, mechanomyography, neural network, spinal cord injuries, torque estimation

## Abstract

This study sought to design and deploy a torque monitoring system using an artificial neural network (ANN) with mechanomyography (MMG) for situations where muscle torque cannot be independently quantified. The MMG signals from the quadriceps were used to derive knee torque during prolonged functional electrical stimulation (FES)-assisted isometric knee extensions and during standing in spinal cord injured (SCI) individuals. Three individuals with motor-complete SCI performed FES-evoked isometric quadriceps contractions on a Biodex dynamometer at 30° knee angle and at a fixed stimulation current, until the torque had declined to a minimum required for ANN model development. Two ANN models were developed based on different inputs; Root mean square (RMS) MMG and RMS-Zero crossing (ZC) which were derived from MMG. The performance of the ANN was evaluated by comparing model predicted torque against the actual torque derived from the dynamometer. MMG data from 5 other individuals with SCI who performed FES-evoked standing to fatigue-failure were used to validate the RMS and RMS-ZC ANN models. RMS and RMS-ZC of the MMG obtained from the FES standing experiments were then provided as inputs to the developed ANN models to calculate the predicted torque during the FES-evoked standing. The average correlation between the knee extension-predicted torque and the actual torque outputs were 0.87 ± 0.11 for RMS and 0.84 ± 0.13 for RMS-ZC. The average accuracy was 79 ± 14% for RMS and 86 ± 11% for RMS-ZC. The two models revealed significant trends in torque decrease, both suggesting a critical point around 50% torque drop where there were significant changes observed in RMS and RMS-ZC patterns. Based on these findings, both RMS and RMS-ZC ANN models performed similarly well in predicting FES-evoked knee extension torques in this population. However, interference was observed in the RMS-ZC values at a time around knee buckling. The developed ANN models could be used to estimate muscle torque in real-time, thereby providing safer automated FES control of standing in persons with motor-complete SCI.

## Introduction

Individuals with spinal cord injury (SCI) often require rehabilitation strategies and assistive technologies to facilitate their daily tasks. Functional electrical stimulation (FES) enables these individuals with neuromuscular disability to execute functional activities such as walking, cycling, and standing up, as well as improving their blood flow and sensory awareness (Petrofsky, 2004). FES activates the nerves using small electrical currents, thereby recruiting muscles to produce non-physiologically evoked contractions and retrain atrophied muscles, thereby partially or fully regaining lost functions (Hamid and Hayek, 2008). Electrical stimulation can be applied through the skin surface or via intramuscular electrodes to evoke contractions of the non-innervated muscles (Ferrarin and Pedotti, 2000). The intensity and temporal characteristics of the stimulation must be regulated to prevent rapid-onset muscle fatigue that leads to failure to perform the desired movement.

When an able-bodied individual performs exercise, over time the muscles becomes fatigued due to repetitive muscle activity, and thus are not be able to reach a set level of maximum voluntary contraction (MVC) force to maintain the current task (Barry and Enoka, 2007). The definition of muscle fatigue in an engineering context is when the muscle's physiological performance change before being finally unable to produce any more force (Barry and Enoka, 2007). This can be used as the basis for determining the muscle fatigue threshold whereby a certain percentage from the MVC during an experiment can be used to determine that the muscle has become fatigued. Another parameter that can be used to quantify muscle fatigue is a change of joint angle (Barry et al., 1985; Guo et al., 2008).

Apart from torque and angle measurements, a physical sensor to measure muscle activity and performance is the mechanomyogram (MMG). MMG records mechanical changes of the muscle during its contraction (Weir et al., 2000). Unlike electromyography, MMG does not have power line interference and has high signal to noise ratio (Islam et al., 2013). MMG also provides information such as forces the muscle can produce, the stiffness and the fluid pressure (Barry et al., 1985). MMG signals during specific activities such as walking, standing and reaching are recorded in order to monitor the muscle fatigue by placing the MMG sensors on the skin surface of the muscle to provide a measure of the mechanical activity of contracting muscles by detecting the muscular sound (Islam et al., 2013). The amplitude of the MMG is related to the force produced by the muscle, whereby even a small change of force is reflected in the MMG amplitude (Beck, 2010).

MMG has been used as a development tool to find the abnormalities from the designated baseline. MMG is useful in the detection of muscle fatigue during sustained voluntary contraction (Jensen et al., 1994). Even though MMG has been commonly used to quantify muscle fatigue during isometric contractions, the usability of MMG for postural control after fatigue made it significant in various fields such as occupational therapy and ergonomics (Beck, 2010).

Researchers have not been able to measure muscle performance during activities such as standing because there is no adequate tool to directly quantify knee and hip extensor torques in stance. With the use of MMG, the muscle activity can be quantified over time and thus its performance assessed. Therefore, in this study, the aim was to design an artificial neural network (ANN) that could predict the torque exerted around the knee joint by the quadriceps muscle by taking inputs from certain MMG parameters, namely the root mean square (RMS) and zero crossings (ZC). The models were designed to predict the muscle torque during FES isometric knee extension. Second, we sought to apply the ANN models to multiple sessions of FES standing challenges. This was done to determine the accuracy and reliability of the ANN models based on RMS and RMS-ZC inputs to predict the knee torque produced by the quadriceps in FES isometric knee extension and standing. Finally, this study aimed to compare the ANN model's performance to determine the input(s) that best predicted of performance of isometric knee extension and standing. In other words, the ANN's accuracy to predict knee torque produced by the quadriceps was tested during FES isometric knee extension and the developed model was then deployed in an FES standing activity. It was hypothesized that the knee extension torque could be modeled through MMG-derived RMS and ZC, which would enable the prediction of torque in activities where torque cannot be physically measured, such as upright stance.

## Methodology

The study was performed in three phases, the first being data collection where the SCI participants performed electrical stimulation-evoked isometric knee extensions to obtain their muscle MMG signal parameters and torques. The second phase was ANN model development and signal processing of the captured previously acquired MMG data from the first phase to process the signal as input for the ANN model. In the third phase the ANN models were deployed in FES-evoked standing performed by the SCI participants. In this study, 3 subjects with SCI were employed in the ANN design and 5 subjects with SCI were used for the standing protocol. Subject 1, Subject 2 and Subject 3 test data were used to train and test the ANN in seated evoked contraction while all 5 subjects were used to test ANN model to estimate torque in evoked contraction in standing environment. The study was approved by the University of Malaya Medical Centre Medical Research Ethics Committee [Ethics Number: 1003.14 (1)].

### Phase 1: knee extension training data collection

This experiment was conducted to obtain the mechanical signal and torque during isometric FES contractions of the quadriceps muscle in three SCI individuals. The torque data were recorded with a dynamometer (System 4; Biodex Medical System, Shirley, NY, USA) and the MMG data were recorded using MMG sensor (Sonostics BPS-II VMG transducer, sensitivity 30 V/g). The subjects were asked to repeat the same isometric knee extension protocol in two sessions with 48 h between each. The experiment was conducted at the Department of Rehabilitation Medicine, University Malaya Medical Centre.

The data obtained from the experiment were then used as the foundation to design a neural network system in MATLAB toolbox to predict torques. The neural networks were tested on with the MMG data obtained during the FES standing contraction without torque data in Phase 3. The next phase of the experiment involved training the system and validating the system.

#### Equipment and materials

The validation of the ANN model was done by comparison with isometric knee torque data obtained from the commercially available dynamometer (System 4; Biodex Medical System, Shirley, NY, USA). The test protocol set on the dynamometer was Isometric knee extension and 900 seconds recovery between each trial. Three trials were conducted for each of the left and right leg. The isometric contraction angle was set at 30° from the straight leg position. The subjects for this experiment were three individuals with SCI (International Standards for Neurological Classification of Spinal Cord injury (ISNCSCI) of A and B) who were trained FES users and non-sensate due to the sensory deficit of their injury. The subjects were briefed about the research protocol before providing their informed consent to participate.

#### FES evoked muscle contractions and knee torque measurement

The subjects were familiar with the FES activity and therefore no familiarization session was needed prior to data collection. The FES stimulation of square-wave pulses was provided at 30 Hz and 200 μs pulse durations with a stimulation current amplitude of 100 mA. The stimulation was provided by a commercial neurostimulator (RehaStim™, Hasomed GmbH, Magdeburg, Germany). Electrodes used in this experiment were 9 × 15 cm^2^ self-adhesive electrodes.

#### Data collection procedure

The subjects were seated on the dynamometer seat and seatbelts were strapped around them to prevent movement from muscles other than the quadriceps interfering the reading of the MMG. The knee attachment was applied to the right leg to measure the torque exerted around the right knee. The subject's ankle was strapped to a cushion of the knee attachment to hold the leg at a 30° knee angle. Since the armature prevented the leg from moving, the torque signal obtained from the dynamometer fully originated from the subject's muscle and not affected by the gravity. The maximum and minimum flexion and extension were set on the Biodex. The Biodex recorded knee torque at a sampling rate of 500 Hz.

**Figure 1:**
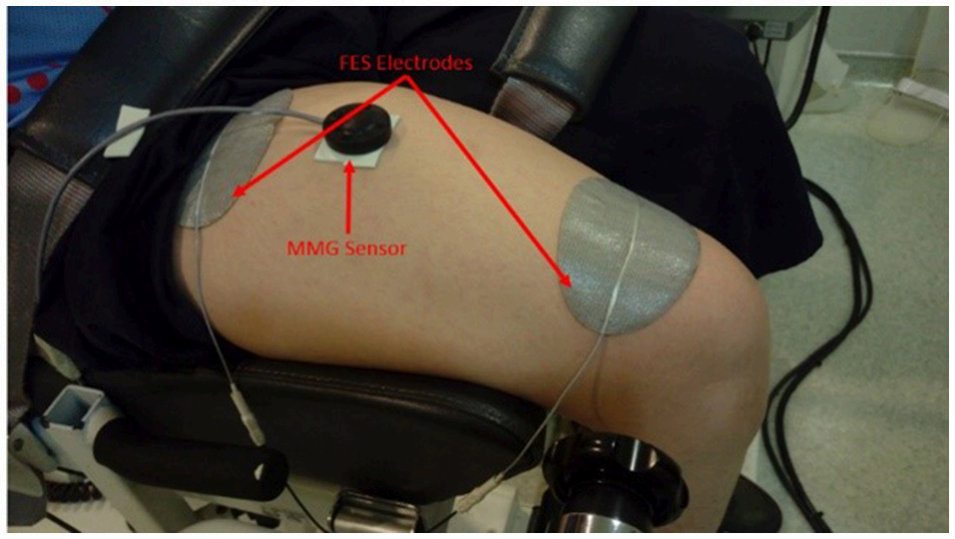
FES electrodes and MMG sensor placement on the quadriceps muscle.

The FES electrodes were placed at both ends of quadriceps muscles but not on the tendon area which was around 5 cm near the position of the patella and around 8 cm distal to the groin area (Levin et al., 2000). Figure 1 illustrates the setup for FES induced isometric knee torque measurement. The subject was seated on the Biodex seat such that the lateral femoral condyle was parallel to the dynamometer axle. This body position and the lever arm of the dynamometer were consistent throughout the study.

Once the settings were set, the dynamometer guided the knee attachment to 30° knee flexion. The MMG recording was initiated first while the dynamometer torque recording and FES stimulation were started simultaneously after. The recording of the dynamometer, the MMG, and the simulation was stopped once the torque reading reached well below 50% of the maximum torque and the recovery period began thereafter. The same procedure was repeated for the left knee once the third trials had ended with the same settings for dynamometer and neurostimulator as well the recovery period. The subject then repeated the same procedure after 48 h. To ensure high day-day reproducibility of the protocol, the same researchers and physiotherapists were involved in the experiment for all subjects.

#### MMG acquisition and processing

Muscle mechanical signals were recorded with the MMG sensor placed right on the muscle belly and held onto the muscle belly with a double-sided tape (3M 157 Center St. Paul, MN, USA). Acqknowledge v4.3 data acquisition and analysis software (MP150, BIOPAC Systems, Santa Barbara, CA, Inc) were used to collect the data at 1 k Hz frequency. The signal was then filtered with a bandpass filter (20 Hz lower cutoff frequency and 200 Hz higher cutoff frequency). The MMG amplitude is a recognizable way to see the relation between MMG and net torque as the decrease of the net torque correlated to decrease of MMG (Gobbo et al., 2006).

The dataset processed from the MMG signal could be in the time or the frequency domain. In the time domain, the amplitude was identified as voltage values and the amplitude was used to calculate RMS. The MMG RMS is reported as a variable in describing motor unit recruitment during a contraction process (Orizio et al., 2003).

The RMS was the magnitude of the measurement obtained by the MMG and the data was in the time domain. Both parameters (RMS and torque) were then scaled to values in the range of 0–1 to simplify the data for preprocessing step for the ANN. The MMG RMS were obtained from MATLAB at 1 s epochs. Normalization of MMG and torque data, as well as the designing process of the ANN, was done using MATLAB version R2015a (2015) toolbox.

RMS was correlated to load as increasing MVC increased the RMS value of the MMG (Akataki et al., 2003). RMS value represents the motor activation (Weir et al., 2000). RMS has also been reported to be an important parameter to monitor muscle fatigue due to its association with the force of contraction of the muscle (Barry et al., 1985). The equation for the RMS processing was defined as:

(1) $$\begin{array}{r} RMS=\;\sqrt{\frac{1}{N}\sum_{k=1}^{N-1}x_{k}^{2},\;for\;k=1,\ldots ,N} \end{array}$$

where *x*_*k*_ is the raw signal from each segment and N is the number of samples.

In isometric contractions, an increase of MMG amplitude was observed when force production was low which was around 10–40% of the MVC. During high level of muscle torque which was around 50–80% of MVC, there was no change in MMG amplitude (Perry et al., 2016). The same observation was reported by another research group (Rodriguez-Falces and Place, 2013).

A lower level of muscle torque resulted to decrease in MMG amplitude (Orizio et al., 2003) due to a linear relationship reported between the contraction muscle and the RMS amplitude of the MMG (Oster and Jaffe, 1980). The correlation of amplitude of MMG signal and motor unit activation was reported during a voluntary contraction as well as FES contraction (Beck, 2010).

The mean frequency shows the frequency feature of the MMG (Cescon et al., 2004). Zero crossing (ZC) was used due to the fact that unlike mean frequency, ZC does not require the use of Fast Fourier Transform (FFT) to obtain and the calculation used to obtain ZC is a simple one (Hägg, 1991). ZC has been defined as the number of times that the MMG signal passed through the horizontal amplitude axis (Zecca et al., 2002). The Equation (2) for ZC was as follows:

(2) $$\begin{array}{l} \;ZC=\;\sum_{k=1}^{N}sgn\left(-x_{k}x_{k+1}\right),\;for\;k=1,\ldots N\\ sgn\left(x\right)=\{\begin{array}{c} 1\;if\;x>0\\ \;0\;otherwise \end{array} \end{array}$$

where *x*_*k*_ is the raw signals of the of the segment and N is the number of samples.

Both MMG RMS and ZC were taken at the sample rate of *N* = 1,000. While the torque data from the Biodex were averaged to get the mean torque for every 500 torque samples. This was done to obtain the reading of torque, MMG RMS and ZC for every second during the stimulated contraction for synchronization.

### Phase 2: neural network development

#### Training data processing and neural network development

The Neural Network system was designed using MATLAB 2015 using the Neural Network fitting toolbox. The ANN system takes MMG inputs to predict the onset of muscle fatigue with the output of normalized torque ranging from 0 to 1. Two types of ANN models were developed based on the two types of data sets used to train the model, the first was normalized MMG RMS only and the second type was normalized MMG RMS together with normalized MMG ZC; i.e., RMS-ZC. RMS and RMS-ZC were used as the input for the neural network training and the normalized torque was used for the target data for the desired output of the network. The ANN was trained by feeding the RMS and RMS-ZC signals along with the desired signal data, i.e., the torque output from the Biodex, to the models. The samples obtained from the first session from the 3 subjects were used as training samples. Based on a priori correlation test considering 0.91 correlation of probability, alpha error probability of 0.05, 0.2 beta error probability, and 0.84 effect size, at least 6–8 samples were minimally required statistically thus this study employed 18 training data and 12 testing samples of various sample size to test the accuracy of the neural network system. The testing samples were obtained from the second session of the experiment for two of the subjects. The samples were arranged in matrix row. A feed-forward network with sigmoid hidden neurons and linear output neurons was used for the development of the ANN. Sigmoid transfer function was utilized as a transfer function due to the transfer function introduced non-linearity to the network's calculations as well as it is a simple derivative function (Calcagno et al., 2010). The type of ANN model developed was the multi-layer Perceptron which contained multiple layers of computational units that were interconnected in a feed-forward manner. The three layers used were the input layer, hidden layer and the output layer. ANN model training technique involved the output values of the system to be compared with the correct values thus producing the error between the output and the correct answer are computed in an error function (Calcagno et al., 2010). Adjustments were made to the weights on every connection to obtain a smaller value of error function. The percentage of testing data was set at 70% training samples, 15% validations samples and 15% testing samples. These were the default settings for ANN. The number of hidden neurons was set at 10. The number of hidden neuron was chosen based on the number of hidden neurons that gave the best results of the training data (*r* > 0.8) The network was trained with the Levenberg-Marquardt algorithm (Levenberg, 1944):

(3) $$\begin{array}{rcl} w & = & w+\Delta w \end{array}$$

(4) $$\begin{array}{rcl} w & = & {\left[J^{T}J+\;\mu I\right]}^{-1}J^{T}e \end{array}$$

(5) $$\begin{array}{rcl} e & = & R-z \end{array}$$

where *w* was the weight vectors, Δ*w* was the differences between the weight vectors, *J* was Jacobian matrix that included the first derivatives of the network errors according to the weight, μ was a scale parameter, *I* was the identity matrix, *R* is the vector of measured torque, *z* is the vector of predicted torque, and *e* is a vector of the network errors. Post neural training, the network was deployed with the MATLAB compiler and Builder tools to generate a MATLAB function. The training and testing data sets for ANN building can be found at these repositories: figshare | figshare.

#### Neural network accuracy test

In order to quantify the performance of the two ANN models, a correlation between the predicted torque output and the actual torque output as well as the accuracy of the models were identified. To achieve the objective, the network was tested with all the normalized RMS and RMS-ZC from the second session of the 3 subjects. The output torque was then compared with the actual torque obtained from the Biodex with the “fitlm” function on MATLAB to obtain the correlation (r). A critical point of 50% torque drop was chosen in order to test the accuracy of the ANN model by comparing the time for the actual torque in each test data samples to reach 50% torque drop and the time for predicted torque (RMS and RMS-ZC) to reach 50% drop to determine the reliability of the models to detect a specific torque value. The accuracy was obtained from the equation (6). The results from the Neural Network test with the isometric knee extension was presented in Table 2.

(6) $$\begin{array}{r} 1-\;\frac{|predicted\;torque\;time\;-\;actual\;torque\;time|\;\times \;100}{actual\;torque\;time}\% \end{array}$$

### Phase 3: testing the neural network model in FES standing

#### Standing protocol

**Figure 2:**
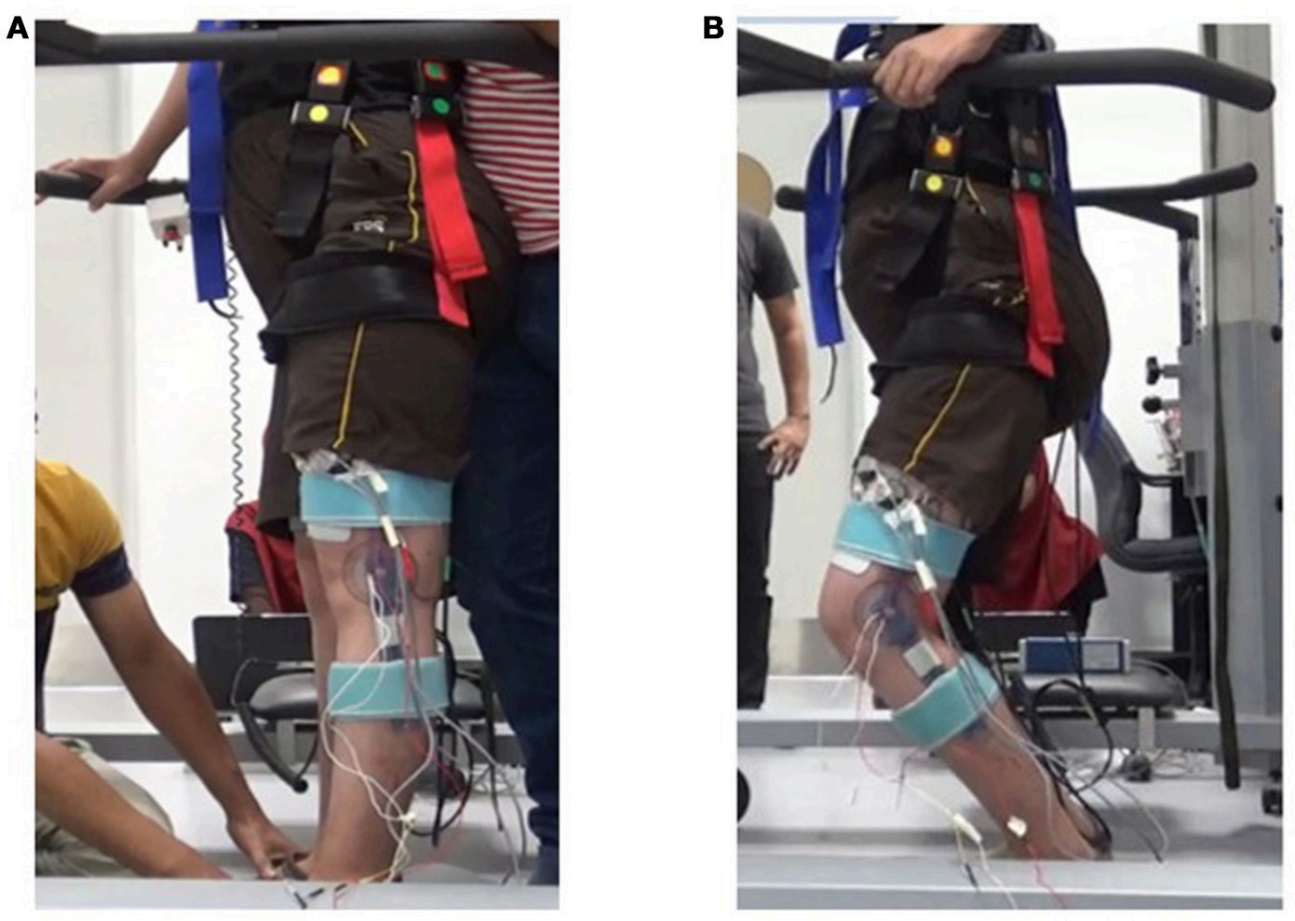
Standing Experiment. **(A)** At the beginning of the experiment the legs were straight due to FES stimulation. **(B)** The knee approaching 30° flexion “knee-buckle”.

A standing protocol was executed in order to validate the effectiveness of the ANN model to predict the onset of muscle fatigue by predicting muscle torque during an FES standing stance in SCI subjects. Five individuals with sensory complete SCI (ISNCSCI A and B) participated in this study phase. This protocol has been developed to measure different stimulation frequency effects during a prolonged FES standing (Ibitoye, 2016, unpublished) and had been approved by the University of Malaya Medical Centre Medical Research Ethics Committee (MECID.NO: 20164-2366). All 5 subjects had been familiarized with the FES training and were able to undergo the stimulation as intended in the protocol. The FES stimulator that was used in the standing experiment was a commercially available neurostimulator (RehaStimTM, Hasomed GmbH, Magdeburg, Germany). The stimulation was channeled to the targeted muscle by 9 × 15 cm^2^ surface adhesive electrodes (RehaStimTM, Hasomed GmbH, Magdeburg, Germany). This protocol was adapted from the procedure reported by Braz and colleagues (Braz et al., 2015). A harness (Biodex Offset Unweighing System) was used to support the subject's body and prevent the subject from swaying and tumbling. Handle bars were available on the subject's sides for upper body balancing. This is because the torque generated by FES was sufficient to maintain the balance of the lower limbs. However, to stabilize the upper body trunk the SCI subject had to hold on to the handle bars to maintain balance due to lack of abdominal and chest voluntary strength. To ensure that the harness did not influence the subject's weight, researchers ensured that both subject's feet were flat on the ground and their heels not hanging above the ground. The muscle mechanical signal during the standing protocol was recorded with the same MMG accelerometer used in the knee extension experiments. Data acquisition and signal processing were done digitally through Acqknowledge v4.3 software (MP150, BIOPAC Systems, Santa Barbara, CA, Inc). FES standing was achieved by continuous stimulation of both left and right quadriceps and gluteal muscles. The quadriceps muscles were stimulated to achieve stabilization in the knee extension and glutei was stimulated for hip extension and upright posture stabilization. The subject was stimulated at quadriceps (80 mA) and glutei (64 mA) at 200 μs pulse width. The frequency of the stimulation was 35 Hz on the one trial and 20 Hz on the other trial. During the stimulation, the changes in the knee bend were observed and verified using a goniometer. The goniometer was used as to identify the end-point for the experiment as the stimulation and MMG recorded was then stopped when the knee reached 30° flexion. The subject was then given a 30-min recovery period between the two trials. The MMG signals obtained from the standing protocol was processed similarly to the signal processing in isometric knee extension. Figure 2 illustrates (a) the setup for the experiment and (b) the moment where the subject was approaching the fatigue point which was the 30° knee bend.

The filtered MMG signal data was then processed to obtain the normalized RMS and ZC. The time taken for the RMS to drop to 70, 50, and 30% of the maximum RMS was taken for *t*-test comparison with the time taken for the knee bend to reach 30°. This was to determine if the RMS alone was sensitive enough to the changes in torque to maintain the knee angle above the 30° mark. The RMS and RMS-ZC data set were then used as inputs for the ANN models respectively to obtain the predicted torque.

A point where changes in the gradient of the predicted output had been selected as a critical point from both sets of predicted torque to determine the consistency between both models to predict the critical point at a similar time and predicted torque value. The time taken to the critical point was normalized in the range of 0–100% stimulation time for all subjects because the overall experiment time differed for each trial and the torque value at the critical point from each standing subject were used in *t*-test to determine its significance. In order to determine the effectiveness of the ANN to predict muscle torque and to compare between the two types of input, few hypotheses had been established to determine the behavior of ANN in standing protocol was similar to isometric knee extension.

The hypotheses were (i) the initial torque predicted would be higher than the final torque predicted, (ii) the predicted torque output pattern would be reduced throughout the stimulation and (iii) the pattern of RMS and ZC before and after the 50% torque drop point would not be the same. To confirm the hypotheses, *t*-test was used to identify the P values of the following pairs; Initial and Final predicted torque, the gradient of MMG RMS and MMG ZC before and after the point where the ANN models predicted a 50% torque drop where there should be a noticeable change to the gradient of MMG RMS and MMG ZC once the predicted torque from each model had reached a 50% torque drop from the maximum, and the gradient of the predicted torque. The statistical analysis was done using PSPP (1.0.1, GNU operating system, 2017). The results from the *t*-test for consistency test for both models are presented in Table 3 while the hypothesis testing results are summarized in Table 4.

## Results

### Testing the ANN model with isometric FES contraction to predict torque

**Figure 3:**
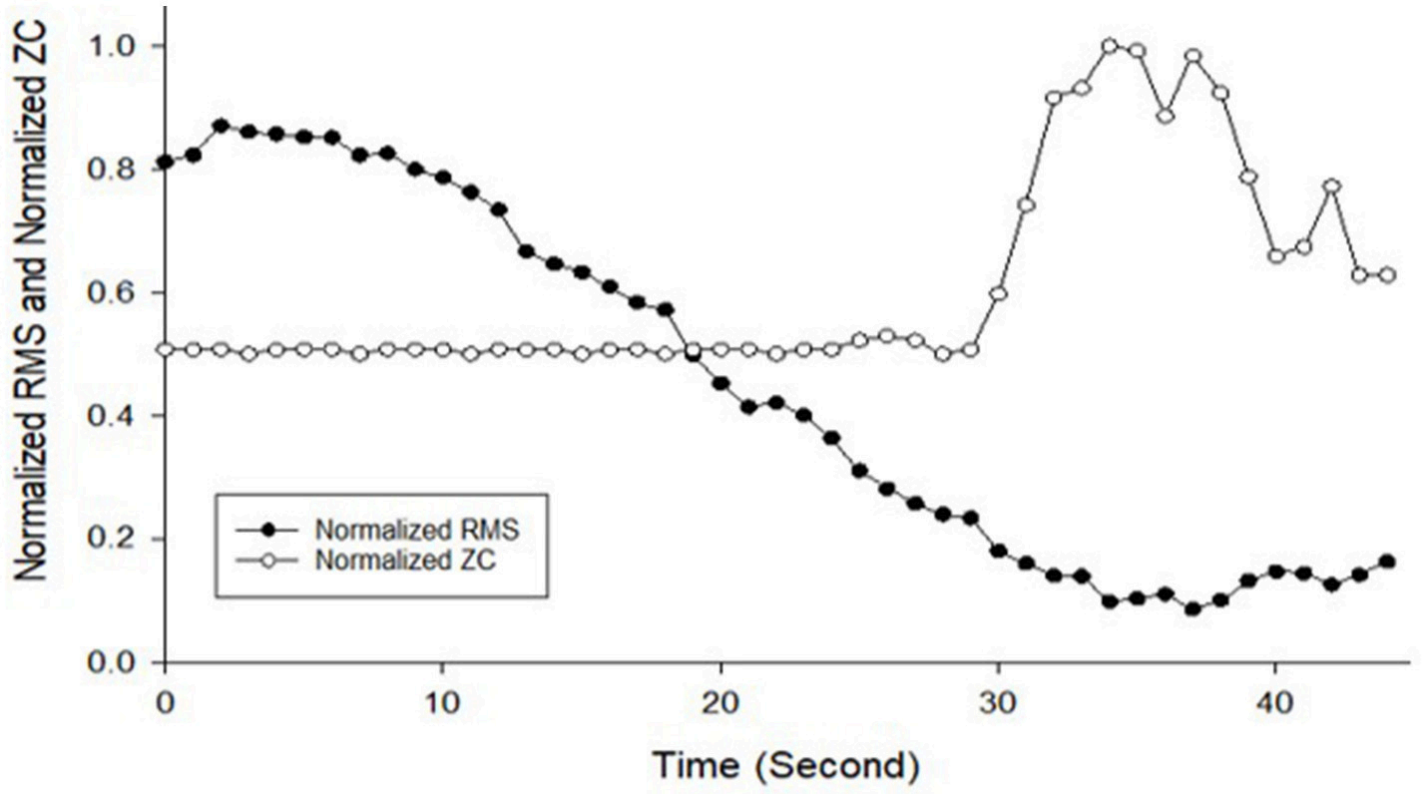
Normalized MMG RMS and Normalized MMG ZC against time used to be as training data for ANN development from Subject 4 Session 1, Left leg trial 1.

**Figure 4:**
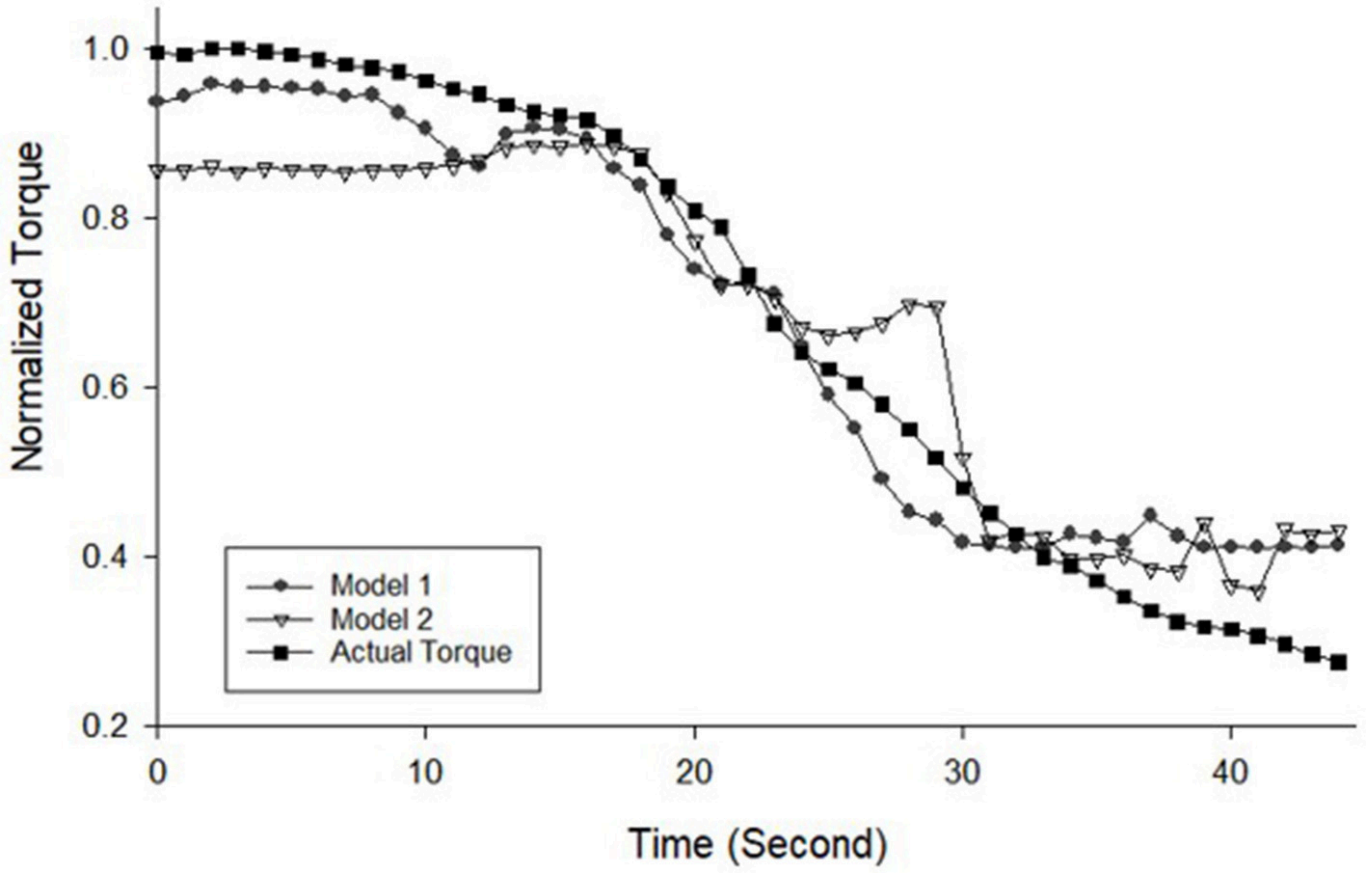
Normalized torque measurement from Biodex dynamometer and the predicted torque from two ANN models from Subject 4 Session 1, Left leg trial 1.

The MMG data were processed into MMG RMS and MMG ZC and then normalized. The final MMG dataset is presented in Figure 3 while Figure 4 illustrates the predicted output torque produced by the neural network model and the actual output torque measured by the dynamometer during the data collection part of the research where Model 1 is the ANN model that uses RMS as input and Model 2 uses RMS-ZC as input. Figure 3 shows RMS gradually decreased from the maximum as the stimulation continues and ZC shows a dramatic increase in the frequency of muscle contraction after a certain period toward the end of the session. The gradient of the RMS decrease differed from the start and toward the end of the contraction.

#### Actual torque and predicted torque from isometric contraction testing

The accuracy of the ANN model to predict the measurement of torque was first tested on isometric knee extension prior to the standing experiment. The correlation and the accuracy of the ANN model to predict the torque in both subject 1 and 2 as presented in Table 1 which shows the mean accuracy and correlation between the two types of inputs.

**Table 1:** Average correlation (R) and accuracy test for the two ANN models to predict torque during FES isometric knee extension.

	**ANN model input**	
	**Model 1: RMS**	**Model 2: RMS-ZC**
R	0.87 ± 0.11	0.84 ± 0.13
Accuracy (%)	79 ± 14	86 ± 11

### Testing the ANN model in FES standing protocol to predict torque

A series of 2-tailed *t*-test was performed to determine whether the time taken for MMG RMS to drop to a certain level was significantly different than the time taken for the knee angle to reach 30° at the end of stimulation. The results from the *t*-test are presented in Table 2.

**Table 2:** *T*-test significance values for time to reach 30, 50, and 70% of MMG RMS drop compared to the time to 30° knee buckle.

**MMG RMS %**	***p*-value**
30% MMG RMS drop vs. 30° knee angle	0.01
50% MMG RMS drop vs. 30° knee angle	0.01
70% MMG RMS drop vs. 30° knee angle	0.02

**Figure 5:**
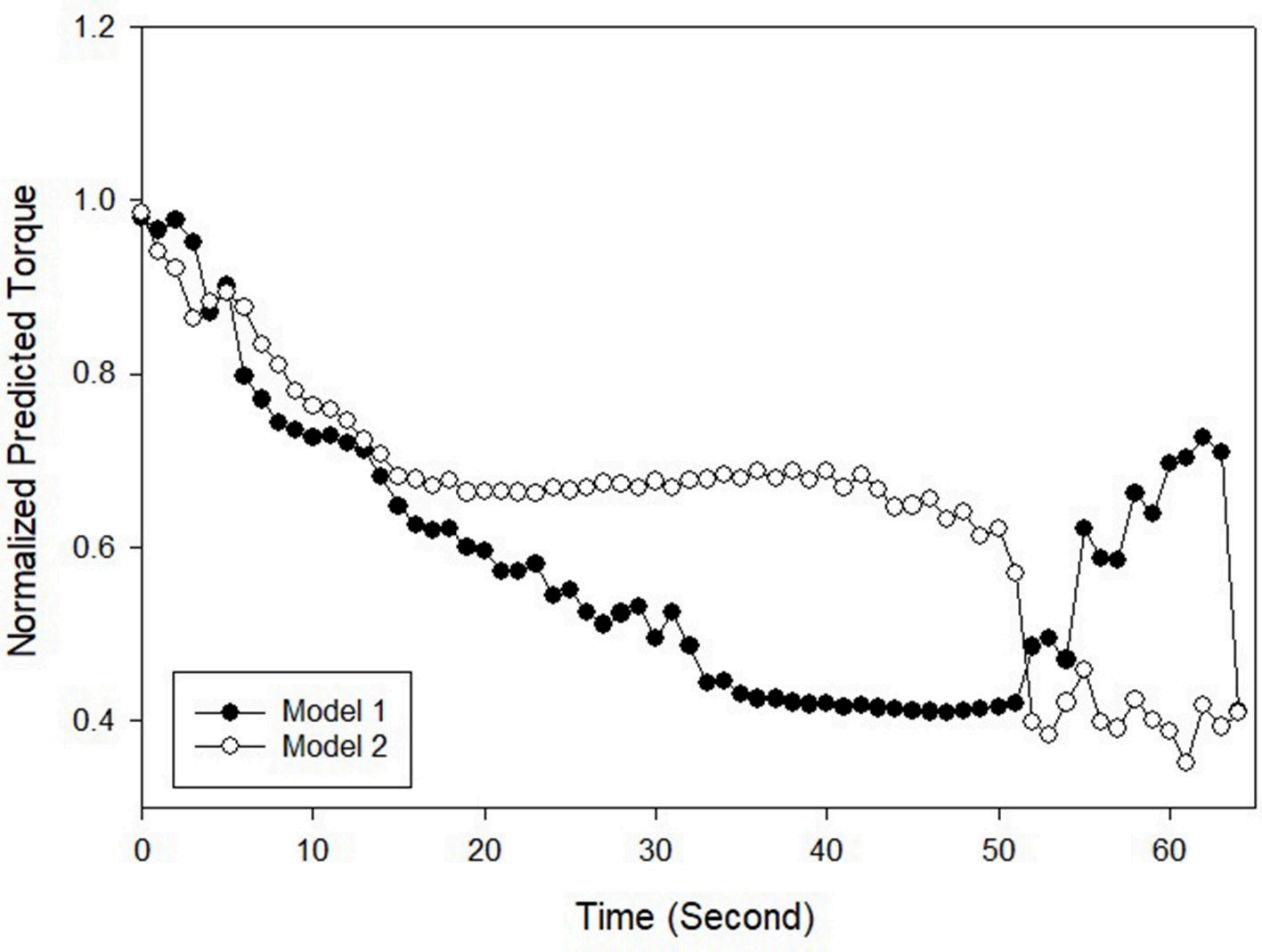
Normalized predicted torque for a standing protocol for Subject 5 Trial 1.

Figure 5 shows the predicted torque, which was the output from the ANN model, where model 1 was based on RMS as input and model 2 was from the RMS-ZC input. Both torque series mostly satisfied the set hypotheses where (i) the initial predicted torque was higher than the final predicted torque, (ii) the predicted torque output pattern descended throughout the stimulation in most cases, and (iii) the gradient of RMS and ZC before and after the 50% torque drop point were different.

The results from *t*-test statistical analysis of the standing protocol based on the said hypotheses are shown in Tables 3, 4.

**Table 3:** Summary of the *t*-test done for time to reach a critical point (RMS and RMS-ZC) and the predicted torque at a critical point (RMS and RMS-ZC).

**Critical Points at which gradient changes**	**ANN model input**		***p*-value**
	**Model 1: RMS**	**Model 2: RMS-ZC**	
Normalized time (%)	44 ± 21	45 ± 17	0.93
Predicted torque (%)	54 ± 14	58 ± 17	0.33

**Table 4:** Summary of t-test statistical analysis for standing protocol from devised hypotheses.

**Hypothesis**	**Torque initial vs. Torque final**		**RMS gradient before and after 50% torque drop**		**ZC gradient before and after 50% torque drop**		**Predicted torque gradient before and after 50% torque drop**	
Model Input	RMS	RMS-ZC	RMS	RMS-ZC	RMS	RMS-ZC	RMS	RMS-ZC
Mean ± SD	Initial Torque		Pre50% drop					
	0.95 ± 0.4	0.93 ± 0.1	−0.02 ± 0.1	0.00 ± 0.00	0.00 ± 0.00	0.00 ± 0.00	0.01 ± 0.10	0.01 ± 0.00
	Final Torque		Post50% drop					
	0.53 ± 0.16	0.51 ± 0.15	0.00 ± 0.00	0.00 ± 0.00	0.00 ± 0.00	0.00 ± 0.00	0.00 ± 0.00	0.00 ± 0.00
*p*-values	0.00	0.00	0.00	0.04	0.18	0.66	0.00	0.01

## Discussion

This study sought to investigate the practicality of using ANN models to predict the knee extension torque during isometric contraction and standing stance using RMS and RMS-ZC as inputs to the ANN. The testing on isometric knee extension revealed that the ANN model used to predict muscle torque from the MMG muscle signal of the quadriceps muscle was reliable. RMS-ZC input ANN model revealed a higher accuracy compared to RMS input ANN model which suggested that in isometric knee extension, RMS-ZC was more suitable than RMS only as input to the ANN model. This also suggests that ANN is a feasible strategy to predict torque without the need of dynamometer. However, when frequency of the stimulation is increased, the initial frequency of the MMG would also increase. This can be seen in Figure 3, whereby when the muscle is fatigued there is a rise in the initial frequency.

The effect of pulse width on the MMG or fatigue was not studied in this research, however other literature suggested that the pulse width has no significant effect on the muscle fatigue but it affects the maximum muscle force production (Jailani and Tokhi, 2012).

Higher accuracy from RMS-ZC input was due to an increase of ZC value past ~50% of maximum knee torque. This was due to SCI muscle are more fatigable compared to able-bodied especially during low-frequency FES (Mahoney et al., 2007). This could be explained by the transformation of slow twitch muscle fiber to fast twitch muscle fiber (Bickel et al., 2004). The transformation explains the ZC graph where the increase of the number of contraction leads to decrease of torque recorded by the dynamometer.

From Table 3, the *t*-test results of *P* = 0.93 indicated no significant difference between the time taken for the predicted knee torque output pattern to reach the point where there are significant changes to the pattern of the actual knee torque obtained from the Biodex dynamometer. The value of the predicted torque at the critical time from both models were not significantly different from the value of the torque obtained from the dynamometer with a *p*-value of 0.33. This indicated that in general both models performed with a consistent level of prediction.

Individually, for the first hypothesis in the standing protocol which states that the initially predicted torque was significantly different than the final predicted torque, both RMS input and RMS-ZC input ANN model outcome revealed that they are significantly different (*P* < 0.01). The difference was due to the rapid muscle fatigue which lead to decrease of RMS and an increased frequency of muscle contraction based on the findings in isometric knee extension (Barry et al., 1985).

The second hypothesis which stated that at the point where the ANN predicted 50% quadriceps torque or lower, there was a significant change toward the pattern of RMS where the RMS decreases at the steeper slope and ended up plateauing (gradient is near 0). However, *t*-test for prediction for both RMS input and RMS-ZC input for the gradient of ZC before and after the predicted 50% torque drop shows that there is no significant difference (*P*-value_rms_ = 0.18, *P*-value_rms−zc_ = 0.66). When compared to isometric knee extension protocol, the standing protocol did not stabilize the legs and this caused the legs to move and this movement had possibly caused the changes in amplitude in the ZC value.

The third hypothesis was that the gradient of predicted torque for both models of ANN is decreasing throughout the experiment. The RMS input showed a slightly more significant difference compared to RMS-ZC input. Although from Table 3 both models showed the same consistency in predicting the torque generally, RMS input showed better reliability in predicting muscle fatigue compared to RMS-ZC input due to less disturbance to RMS when there is a leg movement. However, ZC input was able to provide a frequency domain of the muscle contraction as an increased number of contraction indicated the recruitment of fast twitch muscle fiber which had less endurance to fatigue compared to slow twitch fiber (Karlsson et al., 1981). Additionally, as shown in Table 3 there was significant difference between the time taken for RMS MMG to record a drop to selected level and the time for the muscle to get fatigued and unable to maintain quiet standing. This assumption enabled the ANN to be more useful in predicting the torque at higher accuracy. With both ZC and RMS a better model can be developed that combines both temporal and spectral domain of the muscle signal.

**Figure 6:**
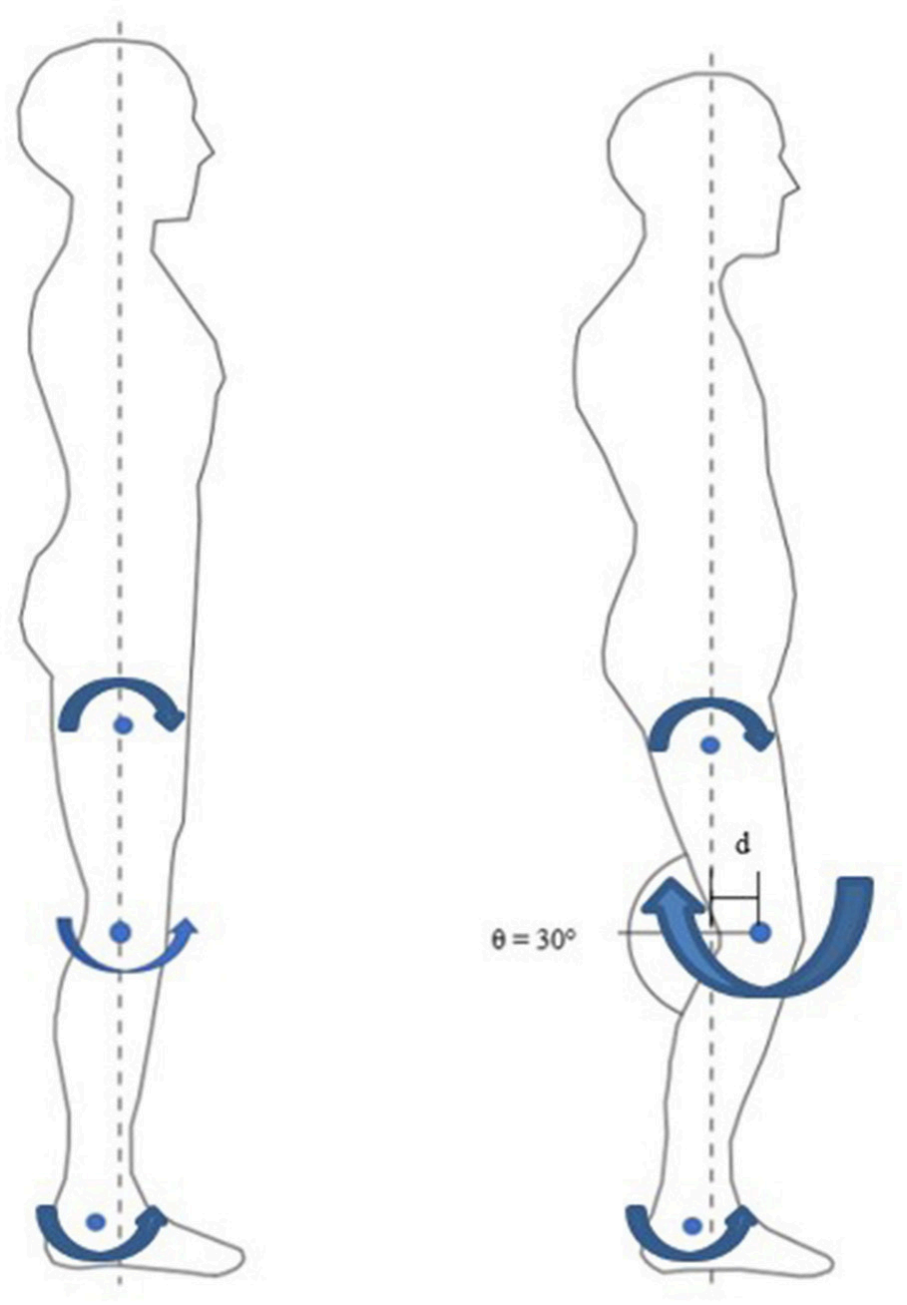
Biomechanics of Standing. Left: non- fatigued, quiet standing motion. Small knee extension moment. Right: fatigued, 30° knee angle bend. Large knee flexion moment due to gravity.

**Figure 7:**
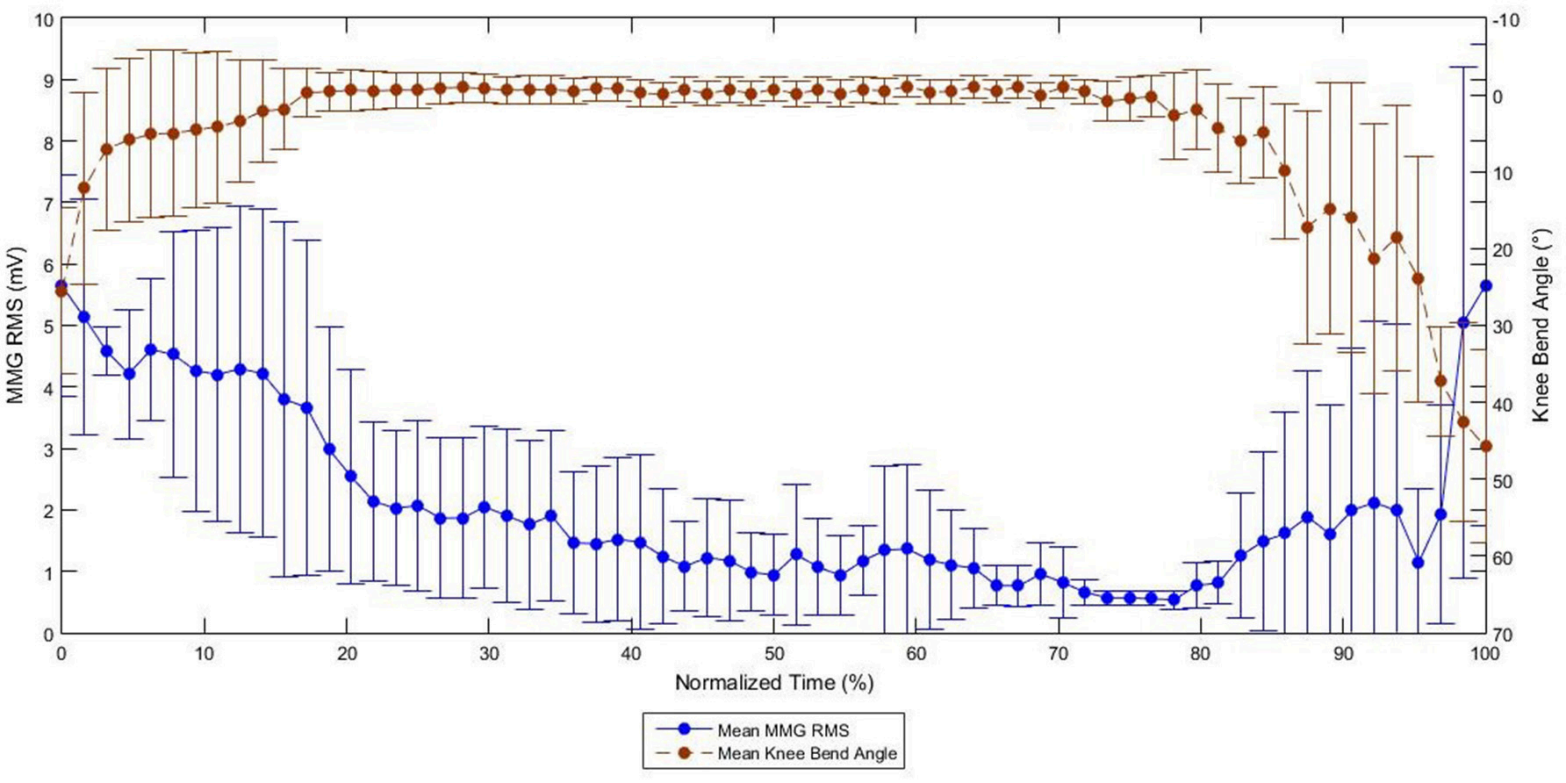
Graph of MMG RMS against Knee Bend Angle during FES Standing in SCI subjects (Mohd Rasid, 2017).

At the end of the evoked standing session, the irregular torque predicted by the models, as illustrated in Figure 5, could be due to gravity effect acted during standing. The biomechanics of standing is illustrated in Figure 6. We hypothesized that the amplified torque due to the gravity and the increased distance (d) between the knee joint and the ground reaction force had affected the MMG responses. A research done with similar protocol and SCI subject supported this hypothesis whereby when the knee started to buckle, the MMG amplitude started to increase. The graph from the experiment is shown in Figure 7 (Mohd Rasid, 2017). However, a biomechanical study which include the study procedure involving biomechanical setup such as ground reaction force plate and a 3D camera system is required to further ascertain this.

This research was limited as presently the ANN model to predict the torque was analyzed only during quiet standing and isometric knee extension. Future studies should include a wider movement pattern such as sit-to-stand movement, which is another nonmeasurable knee torque movement. Different types of inputs such as PTP and ARV in the time domain and MP in the frequency domain could be investigated as well as different types of computer software networks such as support vector machine (SVM). This research also focused on a specific set of parameters for the FES. To our knowledge, there has not been any investigation on ANN model that is trained to predict torque in FES standing experiment using MMG. Hence, this study has demonstrated that an ANN model is feasible in predicting torque during isometric knee extension and FES standing. We hope that this study will be used as the basis for development of real-time ANN model to predict torque and thus may contribute to the improvement of the automated control FES during rehabilitation in SCI.

## Author contributions

MD wrote the manuscript supervised by NAH and with critical feedback from GD and NH. The experiment procedure was conceived by GD, NH and NAH. Isometric FES contraction was done by MD and the standing protocol was done by MD, NAH, and NH. The signal processing and analysis were done by MD and NAH.

### Conflict of interest statement

The authors declare that the research was conducted in the absence of any commercial or financial relationships that could be construed as a potential conflict of interest.
